# Mpox and oral health among children in Africa

**DOI:** 10.3389/froh.2025.1539833

**Published:** 2025-03-11

**Authors:** Moréniké Oluwátóyìn Foláyan, Adeyinka Ganiyat Ishola, Ahmed Bhayat, Maha El Tantawi, Nadia Adjoa Sam-Agudu, Nicaise Ndembi

**Affiliations:** ^1^ The Africa Oral Health Network (AFRONE), Alexandria University, Alexandria, Egypt; ^2^ Department of Child Dental Health, Obafemi Awolowo University, Ile Ife, Nigeria; ^3^ Department of Nursing, University of Ibadan, Ibadan, Nigeria; ^4^ Department of Community Dentistry, University of Pretoria, Pretoria, South Africa; ^5^ Department of Paediatric Dentistry and Dental Public Health, Alexandria University, Alexandria, Egypt; ^6^ International Research Center of Excellence, Institute of Human Virology Nigeria, Abuja, Nigeria; ^7^ Department of Pediatrics and Child Health, University of Cape Coast School of Medical Sciences, Cape Coast, Ghana; ^8^ Global Pediatrics Program and Division of Infectious Diseases, Department of Pediatrics, University of Minnesota Medical School, Minneapolis, MN, United States; ^9^ Division of Epidemiology and Prevention, Institute of Human Virology, University of Maryland School of Medicine, Baltimore, MD, United States

**Keywords:** child, oral health, monkeypox, Africa, disease surveillance, pandemics, outbreaks

## Abstract

Much like it has historically, recent mpox outbreaks in Africa have significantly affected children and highlight major public health challenges, including oral healthcare in pandemic responses. The global 2022 outbreak saw an unprecedented number of mpox cases outside Africa, however children were a minority, constituting less than 2%, with the vast majority of cases reported among adult men who have sex with men. However, African countries continue to report high proportions of pediatric mpox cases, particularly Clade 1 in Burundi, the DRC, and the Congo, and Clade 2 in Nigeria. Oral manifestations of mpox such as ulcers and lesions on the lips and tongue are common and can precede skin rash, making early diagnosis challenging, especially in low-resource settings. Misdiagnosis is also a risk due to the similarity of mpox oral lesions to common pediatric exanthems and enanthems. Oral and other manifestations so mpox among affected children in Africa may be worsened due to delayed treatment and increased morbidity from high rates of malnutrition and immunosuppression, including due to HIV. This paper explores the implications of mpox on the oral health of children in Africa, emphasizing the need for improved surveillance, early detection, and the integration of the mpox response into existing child health programs. Child-focused clinical and public health research, healthcare worker training, and accessible, child-friendly diagnostic tools will be crucial for mitigating the impact of mpox on this vulnerable population.

## Introduction

On August 13 2024, the Africa Centers for Disease Control and Prevention declared mpox a Public Health Emergency of Continental Security in Africa due to its spread to multiple African countries following a massive outbreak in the Democratic Republic of Congo (1, 2). This was followed by the declaration on August 14, of a Public Health Emergency of International Concern by the Director-General of the World Health Organization (WHO) (3). The primary purpose of declaring mpox a public health emergency of international concern is to catalyze timely, evidence-based action to limit the public health and societal impacts of emerging and re-emerging disease risks while preventing unwarranted travel and trade restrictions (4). As at 25 November 2024, over 56,700 suspected cases, and 1,142 deaths, reported from 20 countries in Africa (5).

### Mpox epidemiology in children in Africa

Of concern during public health emergencies is the likelihood that vulnerable populations like children may experience delayed and/or inadequate attention (6). Mpox is endemic largely in West and Central Africa (Cameroon, Central African Republic, Cote d'Ivoire, Gabon, Liberia, Nigeria, the Democratic Republic of the Congo, Sierra Leone, and South Sudan) (7). Since the first clinical case was reported in an infant in the Democratic Republic of the Congo in 1970, mpox in Africa has disproportionately affected children under 15 years of age (8) with the risk of death highest among children under 5 years (9–16). Prior to the 2022 global outbreak, children under 18 years comprised up to 80% of all mpox cases reported to the WHO; during the first year of the 2022 outbreak, children still comprised approximately 40% of African cases (15). In addition, transmission occur between caregivers and children, as well as intra-uterine transmission between mothers and their children (17).

The WHO declared the 2022 global mpox outbreak over on May 10, 2023. However, in late 2023, there were reports of new clusters of cases in the Democratic Republic of Congo later identified to be cause by a novel monkeypox virus strain called Clade 1b (18). The novel Clade 1b virus appeared to be more amenable to human-to-human transmission than Clade 1a strains historically reported in the Democratic Republic Congo that affected children even more disproportionately.

During the current outbreak from January to May 2024, 70% of reported cases occurred in children under 15 years of age. Moreover, children accounted for 83% of total fatalities, with the highest mortality observed among infants under 1 year old, who had a case fatality ratio of 8.7%. This rate is more than three times higher than the 2.4% case fatality ratio recorded in individuals aged 15 years and older (18). In the Democratic Republic of the Congo, 47.7% of the cases reported between January 1, 2022, to August 18, 2024 were in children 15 years of age or younger (19). Between January 1, 2010 and December 31, 2023, children in the country younger than 5 years had had the highest incidence rate (46.38 per 100,000) and case-fatality ratio (6.0%) (20). We provide more details of Clade 1b infection in African children in the later section.

### Clinical manifestations of mpox in children

Among children, mpox is primarily characterized by a rash that progresses through several stages: macular, papular, vesicular, and pustular, eventually leading to scabbing and desquamation. The lesions are firm or rubbery, well-circumscribed, deep-seated, and often become umbilicated in later stages. The rash can be localized or widespread, affecting different body areas, including the palms and soles, at different times. Lesions are typically painful or itchy. Additional symptoms could include fever, chills or sweats, sore throat, headache, myalgias, and lymphadenopathy (21). Most pediatric complications arise from secondary bacterial infections, including encephalitis, pustular corneal lesions, corneal scarring, submaxillary abscess, retropharyngeal abscess, and bronchopneumonia (21).

### Oral manifestations of mpox

Oral manifestations are common in mpox, affecting nearly 70% of clinical cases (22), and 96% of laboratory-confirmed cases (23). Lesions, often preceding skin rashes, may be severe enough to require hospitalization (24), and commonly affects the lips and tongue (25). They include ulcers, pustules, perioral erosions, candidiasis, and oropharyngeal lesions (25). A meta-analysis estimates oral features range from mouth sores (24.80%) to mouth rash (17.99%) (26). Despite their prevalence, documentation in children remains poor (27).

A rapid review of the literature on oral lesions associated with mpox is highlighted in Table 1. The literature search was conducted in Pubmed, Medline, Research4life, EBSCO and AJOL utilizing terms (“Monkeypox” OR “Mpox”) AND (“oral lesions” OR “oral symptoms” OR “mouth ulcers” OR “oral manifestations”). Boolean operators, MeSH terms, and wildcards were used to maximize search sensitivity. The search was conducted for the last 20 years (January 1, 2004–September 30, 2024). The search was for patients of all ages diagnosed with mpox who had oral manifestations. The review included case reports, case series, observational studies, and reviews. The 44 records identified were imported into Rayyan, one duplicate removed, titles and abstracts screened and full texts and their references reviewed. Articles that could not be accessed were excluded. Fourteen articles reported on oral lesions associated with mpox. The data on study location, study design, sample size, and study findings were extracted and collated into a summary as shown in Table 1 (28–40).

**Table 1:** A rapid review of studies reporting on oral health workforce and oral health systems strengthening in Africa.

First author and year of publication	Study location	Study objective	Study design	Sample size	Oral health related study findings
Peiro-Mestres et al. 2022 (28)	Spain	Not specified	Laboratory analysis	12	MPX DNA was detected in saliva from all cases, sometimes with high viral loads
Tarín-Vicente et al 2022 (29)	Spain	To investigate clinical and virological characteristics of cases of human monkeypox in Spain	Cohort study	181 patients identified as gay men, bisexual men, or other men who have sex with men, heterosexual men or heterosexual women	78 (43%) participants presented with lesions in the oral and perioral region.
Coppens et al 2023 (30)	Belgium	To verify the value of select alternative specimen types for mpox laboratory confirmation	Laboratory analysis	25 patients with MPXV-confirmed skin lesions	In patients with MPXV-confirmed skin lesions, the diagnostic sensitivity of MPXV PCR was 80% in EDTA plasma, 64% in oropharyngeal swabs, and 88% in saliva. MPXV viral loads were higher in saliva than in oropharyngeal swabs and EDTA plasma
Tan et al 2022 (31)	Canada	To present a case of monkeypox infection	Case presentation	1 patient with monkeypox infection	Mildly tender, 3-cm right submandibular lymph node, and 0.5- to 1.5-cm crusted ulcers with central depression at the upper lip,
Ramírez-Amador et al 2023 (32)	Mexico	To describe the histological and ultrastructural oral findings of mpox	Case presentation	1 patient living with HIV	Shallow ulcer, covered by a fibrinoid membrane, and surrounded by an erythematous halo in the hard and soft palate
Sah et al. 2023 (33)	Not defined	To identify the otolaryngologic manifestations of MPOX across previous and current outbreaks and among endemic and non-endemic regions	Systematic review and meta-analysis	5,952 patients	13% prevalence of oral ulcers [95% CI (0.02–0.30), *I*^2^ = 99%], 6% prevalence of oral exanthem [95% CI (0.00–0.17), *I*^2^ = 99%]. Features that were more prevalent in endemic areas versus non-endemic areas include 15% prevalence of oral ulcers [95% CI (0.02–0.36), *I*^2^ = 99%].
Gandhi et al. 2023 (25)	Nigeria, Sudan, the Democratic Republic of Congo, USA, Portugal, France, Spain, Brazil	To estimate the overall prevalence of oral manifestations among patients with mpox globally.	Systematic review and meta-analysis of 19 studies	4,042 laboratory-confirmed patients with mpox	Presents as oral/perioral lesions, mouth ulcers, mouth sores, tongue sore, mouth/lips/oral mucosa rash, buccal ulcers. The prevalence ranged from 8.52% to 100%. The pooled prevalence of oral manifestations was 36.75% (95% CI: 23.77–50.65). Subgroup analysis revealed a pooled prevalence of 24.80% (95% CI: 8.14–46.32) of mouth sore, and 17.99% (95% CI: 15.66–20.43) of mouth rash.
Pinnetti et al. 2024 (34)	Italy	To report cases of Mpox pharyngotonsillar involvement	Case series	14 patients with Mpox pharyngotonsillar involvement	Polymerase Chain Reaction for Mpox virus was positive in oropharyngeal swab, saliva and serum.
Huhn et al. 2005 (34)	USA	Not specified	Descriptive analysis	34 patients with a confirmed case of monkeypox	Patients with nausea and/or vomiting and mouth sores were associated with a hospitalization duration of >48 h and with having >3 laboratory tests with abnormal results.
Pittman et al. 2022 (36)	Democratic Republic of the Congo	To describe the results of a prospective observational study of the clinical natural history of human monkeypox virus (MPXV) infections	Descriptive analysis	216 admitted patients with confirmed infection	53 (24.5%) had mouth/throat lesions: 5 (16.1%) children <5-year-old, 22 (32.8%) 5–11-year-old, and 26 (22.0%) >12-year-old.
Whitehouse et al. 2021 (37)	Democratic Republic of the Congo	To evaluate differences in incidence, exposures, and clinical presentation of polymerase chain reaction-confirmed cases by sex and age	Descriptive analysis	1,057 confirmed cases	570 (56.0%) had buccal ulcer: 103 (5.8%) of 0–4-year-old, 109 (56.8%) of 5–9-year-old, 164 (56.0%) of 10–19-year-old, 114 (63.7%) of 20–29-year-old, 53 (56.4%) of 30–39-year-old, and 27 (41.5%) of >40-year-old.
Jang et al. 2022 (38)	Republic of Korea	To report the first case of monkeypox in the Republic of Korea	Case report	I bisexual male patient	Perioral erosive lesions covered with black crusts were found.
Patel et al. 2022 (39)	United Kingdom	To characterise the clinical features of monkeypox infection in humans	Descriptive case series	197 patients with polymerase chain reaction confirmed monkeypox infection	27 (13.7%) had oral lesions
Thornhill et al. 2022 (40)	Mexico, Canada, USA, Argentina, Israel, Portugal, France, Spain, UK, Belgium, Italy, Netherlands, Denmark, Germany, Australia, Switzerland.	To describe the presentation, clinical course, and outcomes of polymerase-chain-reaction-confirmed monkeypox virus infections	Descriptive analysis	528 infections diagnosed between April 27 and June 24, 2022, at 43 sites in 16 countries.	7% had anogenital and oral lesions

Table 1 highlights that oral lesions are common in mpox, with prevalence ranging from 8.5% to 100% (25). Features include buccal ulcers, perioral lesions, tongue sores, and mucosal rashes (25, 29, 33, 36–40), often in individuals aged 10–19 years (37). Cases report fibrinoid-membrane ulcers, perioral erosions with black crusts, and erythematous ulcers (31, 32). Severe cases associate oral sores with prolonged hospitalization and abnormal labs (35). Saliva shows higher viral loads and better diagnostic sensitivity than oropharyngeal swabs (28, 35). Oral ulcers and exanthem are more prevalent in endemic regions, with advanced diagnostics revealing unique patterns (33). However, a significant gap in understanding mpox's oral health impacts in children, with most reports from endemic African regions. In the Democratic Republic of Congo, Pittman et al. found mouth and throat lesions in 24.5% of pediatric cases, highest in ages 5–11 years (36), while Whitehouse et al. reported buccal ulcers in 56% of confirmed cases, with age-based variations (37).

This geographical concentration of data suggests that oral health manifestations in children might be underreported or under-researched in non-endemic regions. The lack of studies outside Africa raises questions about potential disparities in disease recognition, reporting, or clinical presentation in different settings. Furthermore, endemic regions may have unique socio-environmental factors, such as higher exposure risks or limited healthcare access, influencing the frequency and severity of oral health manifestations in children.

The distinct focus on endemic areas also highlights the urgent need for global, region-specific pediatric studies. Such research is critical for understanding whether the observed patterns are consistent worldwide or if they reflect localized factors. Expanding the scope of pediatric oral health studies could also contribute to developing targeted interventions, improving diagnostic capabilities, and addressing potential oral health inequities in mpox management for children globally.

### Implications of mpox outbreaks for the oral health of children in Africa

Children in African countries continue to be at high risk of mpox infection, including from Clade 1b virus. Personal communication with Africa CDC indicated that between January and September 2024, 46% of confirmed cases reported in Africa were children less than 15 years. Children less than 15 years constituted 52% of the cases in Burundi, 47% in DR Congo, 37% in Nigeria and 50% in the Congo. The risk of severe mpox is higher among those who are immunocompromised, including from malnutrition and HIV infection (41). Cases of severe mpox have larger, more extensive lesions, particularly affecting the mouth, eyes, and genitals. Complications can also include inflammation of vital organs, such as the heart, brain, or other organs, as well as secondary bacterial infections of the skin, bloodstream, or lungs (42).

The risk of immunosuppression due to malnutrition and HIV are higher for children in African countries. By the end of 2023, an estimated 1.4 million children (ages 0–14 years) were living with HIV globally, with 120,000 newly infected (43). African countries with ongoing mpox outbreaks, such as Nigeria, South Africa, Kenya, Rwanda, Côte d'Ivoire, and Uganda, also have high HIV burdens (44). Advanced HIV disease and low CD4 cell counts are linked to impaired mucosal recognition of the mpox virus, weakened B-cell response, and reduced viral clearance (27). This increases the risk of severe mpox. Mpox can also present as an opportunistic infection or an AIDS-defining illness (45). However, further research is needed to fully understand the host-pathogen interactions of the mpox virus in individuals with immune deficiency due to HIV infection (46). Of concern is that all children younger than five years of age living with HIV are considered to have advanced HIV disease (47). This risk is a concern for African countries, where the burden of new and established HIV infections is still high (48).

Children in African countries additionally face a high risk of immunosuppression due to malnutrition. While global rates of child stunting are declining, the number of stunted children in Africa increased from 54.4 million in 2000 to 61.4 million in 2020, with an estimated 61 million by 2025 (49, 50). Progress in reducing stunting is slow, at 1.5% per year, far below the 5.5% needed to meet global targets (51, 52). East and West Africa, the regions most affected by mpox, also have the highest rates of malnutrition (53). Malnutrition leads to insufficient intake of essential vitamins and minerals, disrupts gut microbial metabolic processes causing chronic low-grade inflammation (54), damages both the innate and adaptive immunity of the host (55, 56), and increases the body's vulnerability to infections including viral infections (57) like mpox (58).

Mpox complicated by HIV and malnutrition creates a challenging clinical scenario, particularly in pediatric patients, due to the compounded effects of the diseases. In addition to HIV and malnutrition causing immune suppression, which weakens the body's ability to fight infections, HIV and malnutrition are also associated with oral lesions, which can further complicate mpox infection. Candidiasis, ulcerations, and gingivitis, which are lesions associated with HIV and malnutrition (59, 60), cause pain and discomfort that impair nutritional intake, worsening malnutrition. In pediatric mpox cases, the presence of these oral manifestations can lead to dehydration, difficulty eating, and a higher susceptibility to bacterial infections. The combined impact of mpox, HIV, and malnutrition significantly heightens the risk of morbidity and mortality in children, emphasizing the need for comprehensive care that addresses both the infectious and nutritional aspects of treatment. Early intervention, supportive care, and multidisciplinary management are critical in improving outcomes for these vulnerable patients.

### Oral features of mpox may delay mpox diagnosis in African countries

Primary lesions of mpox often originate in the oropharynx before manifesting on the skin (61), which can lead to the initial presentation of mpox infection to dentists. Oral mpox lesions may resemble herpetic and other vesicular-bullous lesions, complicating differential diagnosis (25). Herpetic simplex infection is common in children (62). These lesions are typically treated conservatively, with symptom resolution expected within 10–14 days (63). This possible risk of misdiagnosis may increase the likelihood of mpox transmission within the community.

In Africa, it is common for children to present with oral lesions co-existing with painful, self-limiting skin lesions (25, 36, 37). Conditions such as chickenpox, shingles, or herpes, which have both oral and skin features, can increase the risk of mpox misdiagnosis (8, 15). There is currently little known about the differential oral features of mpox in children and adults or the prognostic oral lesion markers for mpox in children. Oral lesions may be a valuable indicator of mpox in low-middle income countries.

### Residence in an African country increases risk for delayed diagnosis and treatment

Although mpox has been documented in Africa since the 1970s, resources for its surveillance, treatment, and prevention have been scarce. Both Clade 1 and Clade 2 mpox infections can affect children and adolescents, potentially leading to significant morbidity and mortality, including hospitalization, visual impairment, critical illness, and death (64). The management of mild ailments like oral and skin lesions are often routinely undertaken by alternative medical care providers like traditional healers and patent and proprietary medicine vendors (private drug retailers) (65, 66). These alternative care providers serve as the first source of care for between 8% and 55% of illnesses occurring among children under five (67) and are a particularly important source of care in rural and lower income communities (68).

The involvement of alternative care providers in Africa's mpox response has been minimal, with their inclusion in outbreak plans and interventions largely overlooked. As a result, the limited focus on frontline carers, including those responsible for children's health and oral health, may negatively impact the quality of care vulnerable children receive during this period. The self-limiting nature of mpox infection, and the absence of easy to administer rapid diagnostic tools that can be purchased in shops like the HIV, malaria, and COVID−19 self-testing tools, further increases the risk for delayed diagnosis and management.

Vaccines and antiviral medications became widely used to prevent and treat mpox for the first time during the 2022 outbreak, but their efficacy in pediatric patients remains unknown (64) and their access in primary health care setting in Africa remains limited. These complexities associated with the appropriate diagnosis of mpox increases the risk of its delayed or non-diagnosis in children in Africa.

### Oral health inequalities, the mpox outbreak and children in Africa

The high rates of mpox among children expose the deep inequalities in access to healthcare and nutritional support, with immunosuppression due to HIV and malnutrition further increasing their susceptibility. The intersection of infectious diseases and socioeconomic factors, where under-resourced communities face multiple, overlapping health crises including challenges in receiving timely and accurate diagnoses presents a challenge for children, their oral health and their quality of life. The exclusion of first-line responders to children's health problems—traditional healers and patent medicine vendors—in the mpox response underscores another layer of inequality—children in rural and underserved areas may not receive accurate information or timely care. Worse still, access to mpox vaccines and antiviral treatments is limited, particularly for pediatric populations in Africa (15).

When the focus of care for children and adolescents with oral health problems is dentists—to the exclusion of pandemic preparedness and first-line respondents, the existing inequalities are further exacerbated, especially in rural and underserved areas (69, 70). Focusing only on oral health professionals for the management of oral health care needs during the mpox outbreak further creates missed opportunities to integrate oral health into broader health systems for appropriate pandemic preparedness and response, and to prioritise oral healthcare needs of children and adolescents during health crises. In addition, focusing on dentists to provide care to children during the mpox outbreak overlooks the current oral health crisis precipitated by the limited access to specialized dental care across many parts of Africa (71). Where there is specialized oral health care in Africa, economic and geographic barriers make access challenging (72). Furthermore, in times of crisis, such as pandemics, first-line health responders play a crucial role in maintaining healthcare continuity. Neglecting their involvement in oral health management reduces the system's resilience to health emergencies and deepens disparities.

Furthermore, the global inequality in vaccine distribution during the mpox outbreak echoes similar challenges seen with COVID-19, where wealthier nations had better access to life-saving interventions. The WHO-pre-qualified Modified Vaccinia Ankara-Bavarian Nordic (MVA-BN) vaccine—currently the most commonly used mpox vaccine globally—was indicated for use for people above the age of 18 years (73), although the was expanded to include it use in individuals aged 12 years and older on 8 October 2024 (74). Recently, the Japanese Government promised to provide around 3.05 million doses of pediatric-indicated, live attenuated LC16 vaccine for children in Africa (75). The vaccine was only granted WHO Emergency Use Listing for use in children on the 17th of November 2024 (74). There is however, little data on the safety and immunogenicity of the LC16 vaccine in immunocompromised individuals (76), with a proviso for the need to screen for HIV and other significant immunosuppression in children before administering the vaccine, because of the risk of adverse reactions.

### Critical next steps

While the threat of mpox to children and adolescents had been hidden in plain sight (77), that of oral health in children and adolescents with mpox had been largely overlooked. Of concern is the high vulnerability that the triad of being a child, a resident in Africa, and the non-prognostic mpox oral lesions poses. This intersection highlights a pressing need for targeted interventions that address both systemic inequities and the specific clinical challenges associated with mpox.

#### Inclusion and capacity-building of lay providers and first responders

Pandemic response policies at both national and continental levels must recognize the vital role of traditional healers and patent medicine vendors, who are key first-line health responders for children in rural and underserved communities. Actively involving them in the mpox outbreak response is essential to closing gaps in healthcare access and ensuring that vulnerable children receive timely care. Capacity-building efforts should focus on managing fever-associated ailments (78) and recognizing oral lesions as a potential indicator of mpox, even in the absence of skin lesions. Previous epidemics like Ebola and pandemics such as COVID-19 have shown that traditional healers and patent medicine vendors play a crucial role in controlling infectious disease outbreaks by being prompt in screening symptoms of fever, having a high index of suspicion for infectious diseases and referring appropriately for management (79, 80). Early engagement and education of traditional healers and patent medicine vendors on the management of mpox, including recognition of the oral features of mpox. This can be achieved through targeted training workshops with visual aids for identifying oral lesions, and protocols for timely referral to healthcare facilities. Similar efforts had been done successfully with HIV (81, 82).

#### Integration of the mpox response with child health programs

In addition, mpox screening, diagnosis and management should be integrated into existing child health programs, particularly those focused on malnutrition and HIV control. This would allow for a coordinated approach to address mpox alongside other health challenges, improving early detection and care delivery for affected children. This may require that surveillance systems for mpox in Africa should improve their focus and reporting on children and include oro-pharyngeal features as indicators of mpox. In addition, healthcare workers, including those in primary care, need to be trained on the oral signs and symptoms of mpox, guidelines for differential diagnosis and reporting mechanisms.

Mpox response policies should also prioritize the development, distribution and administration of mpox vaccines and antiviral treatments for pediatric populations, especially in primary healthcare settings. Efforts should also focus on ensuring equitable access across rural and urban areas, reducing the disparity in care. Community-based education campaigns can raise awareness about mpox prevention, recognizing early symptoms (especially oral manifestations) in children, and seeking timely medical intervention. These campaigns should leverage existing health infrastructure to reach a broad audience, particularly in rural areas.

Mpox diagnostics priorities include the development of affordable and non-invasive diagnostic tools for that can be easily deployed in primary healthcare settings and by alternative care providers like traditional healers and patent and proprietary medical vendors. Oral self-testing kits for screening for mpox, similar to HIV (83, 84) self-test kits, should be developed. This would facilitate earlier detection and treatment, particularly in children who may be asymptomatic (85). The current emergency approval of $10.4 million by the African Union to Africa CDC for mpox outbreak response (86) should include investment in the development and distribution of cheap self-diagnostic non-invasive tools appropriate for use in children. Oral self-test kits for mpox is a possibility to explore as there are oral self-testing kits for HIV and COVID-19 (87, 88).

#### Pediatric mpox research priorities

Research priorities should include identifying oral markers of mpox in children, the differentiating features of mpox-related lesions from other common pediatric infections, and the clinical (including oral) presentation of pediatric mpox in the setting of Clade 1a, 1b, 2a, and/or 2b infection. This will improve diagnostic accuracy and guide appropriate treatment protocols for pediatric patients. In addition, investigating the interactions between mpox, HIV (89), and malnutrition in children is critical. Research should focus on how these conditions collectively impact the severity of mpox and its oral manifestations, and how treatment protocols can be adapted to better serve immunocompromised and malnourished children. Furthermore, there is a pressing need for studies on the safety and efficacy of mpox vaccines and antiviral treatments in children, particularly those who are immunocompromised due to HIV or malnutrition. These studies will help refine vaccination strategies and ensure that the most vulnerable children are adequately protected. Currently, there are no medications approved by the US Food and Drug Administration for the treatment of mpox, and there is a lack of clinical trial data to inform therapeutic decisions for children.

## Conclusions

Evidence suggests that young children are at increased risk for monkeypoxvirus infection in Africa. At the same time, HIV infection and malnutrition further increase susceptibility to severe mpox disease. Limited availability of diagnostics and the presence of oral lesions similar to the oral lesions seen in mpox may delay the clinical diagnosis and prompt management of the disease. These factors are important to consider when designing surveillance activities to control the spread of mpox in Africa. Dentists need to be aware of the oral manifestations to help detect the infection at the earliest possible stage and to avoid contributing to disease dissemination through cross infection.

## Data Availability

Publicly available datasets were analyzed in this study. This data can be found here: not applicable.
